# Laparoscopic Repair of Larrey Hernia: A Case Report and Literature Review

**DOI:** 10.7759/cureus.53917

**Published:** 2024-02-09

**Authors:** Mohammed Malik Bennani, Viviane Thill, Freddy Mboti

**Affiliations:** 1 Digestive Surgery, Brugmann University Hospital, Brussels, BEL; 2 Digestive, Thoracic and Laparoscopic Surgery, Brugmann University Hospital, Brussels, BEL

**Keywords:** morgagni-larrey hernia, hernia surgery, general surgery, laparoscopic surgery, diaphragmatic hernia

## Abstract

Morgagni-Larrey hernia is a rare pathology resulting from an anterior diaphragmatic defect. Diagnosis is often made in adulthood due to the lack of symptoms associated with this condition. Various surgical techniques have been reported for its treatment, but no standard approach has been established due to its rarity. Here, we present the case of a 42-year-old patient with a symptomatic Larrey hernia successfully treated with a laparoscopic approach. The rationale for documenting this case lies in contributing to the understanding and management of this rare condition.

## Introduction

Congenital diaphragmatic hernia (CDH) is a rare malformation, with the posterolateral Bochdalek hernia being the most common type [1]. Morgagni (right-sided) and Larrey (left-sided) hernias represent 90% and 2%, respectively, of anterior hernias, with bilateral hernias comprising the remaining 8%[2]. We report the case of a 42-year-old woman with gastrointestinal and respiratory symptoms associated with a large left anterior diaphragmatic hernia treated laparoscopically. The documentation of this case aims to contribute to the understanding of this condition.

## Case presentation

**Figure 1:**
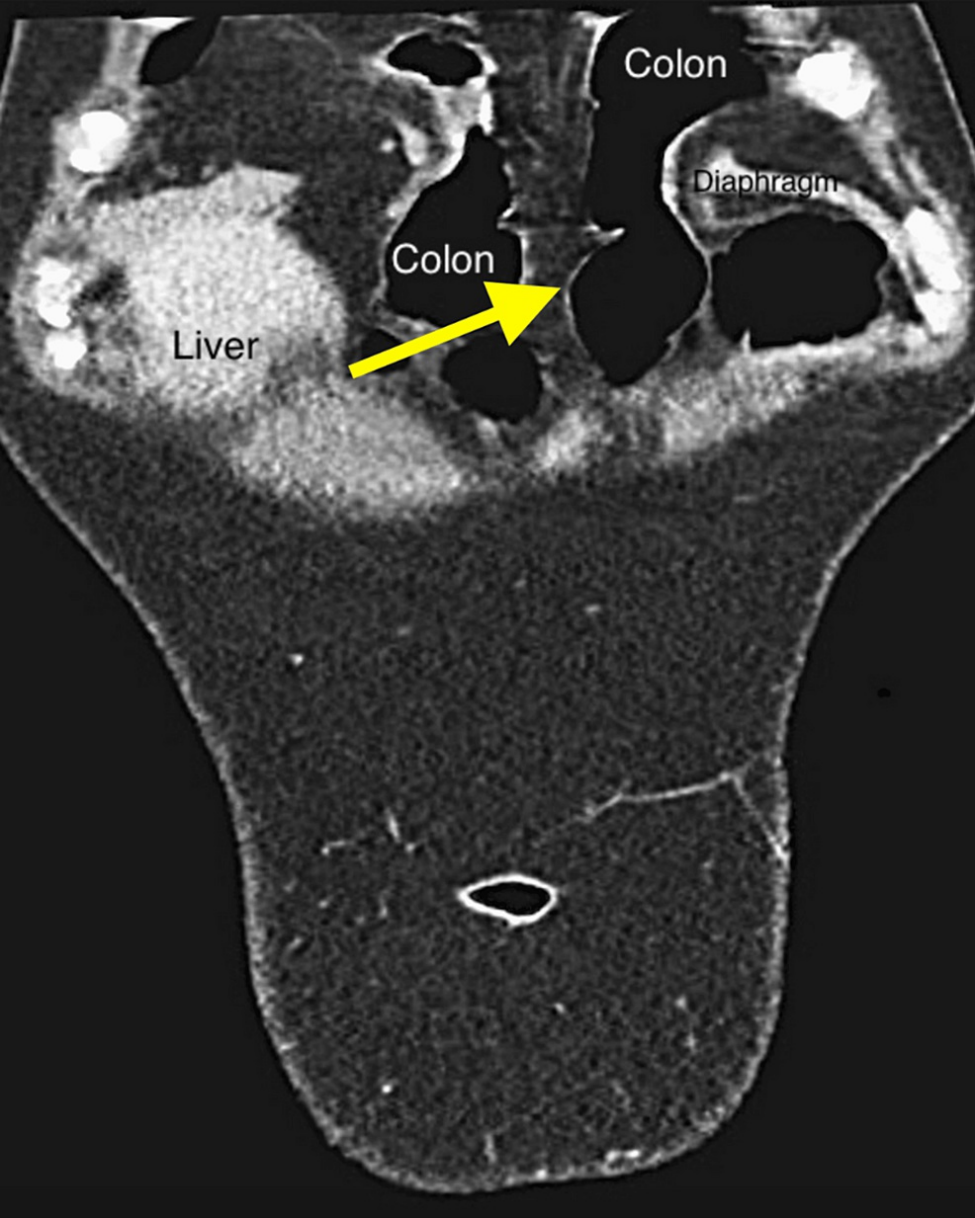
Frontal view of abdominal and thoracic CT scan showing an anterior diaphragmatic hernia containing transverse colon and greater omentum (yellow arrow)

**Figure 2:**
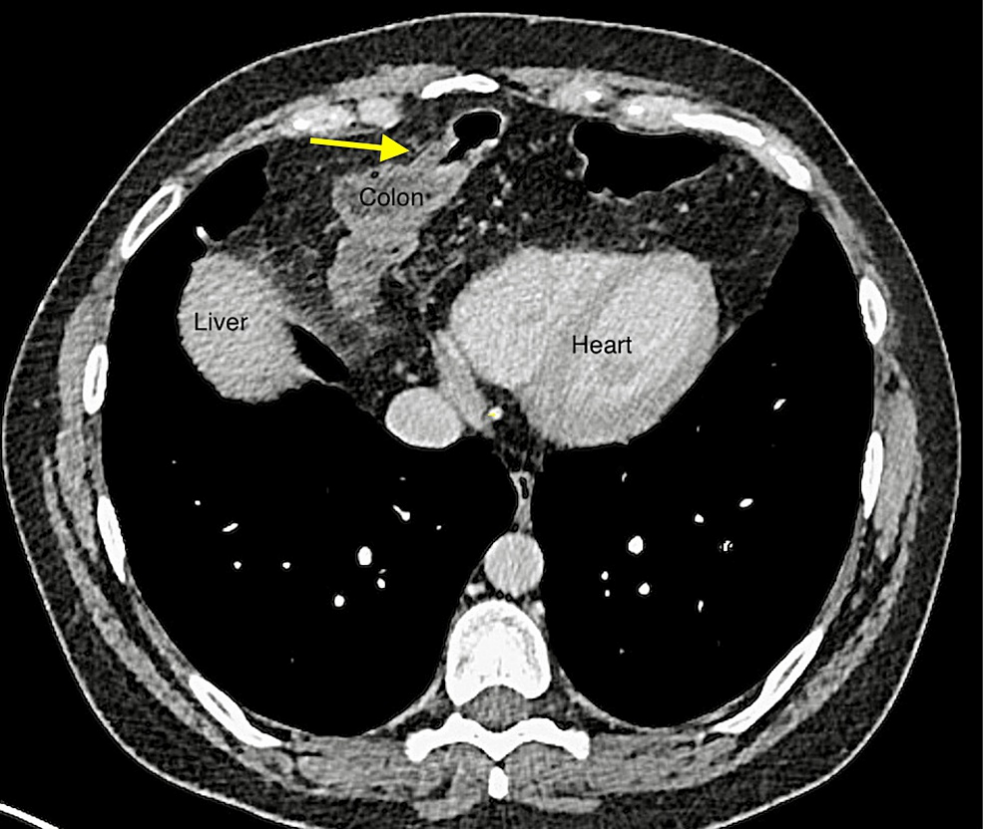
Axial view of abdominal and thoracic CT scan displaying transverse colon and greater omentum in the anterior mediastinum (yellow arrow)

**Figure 3:**
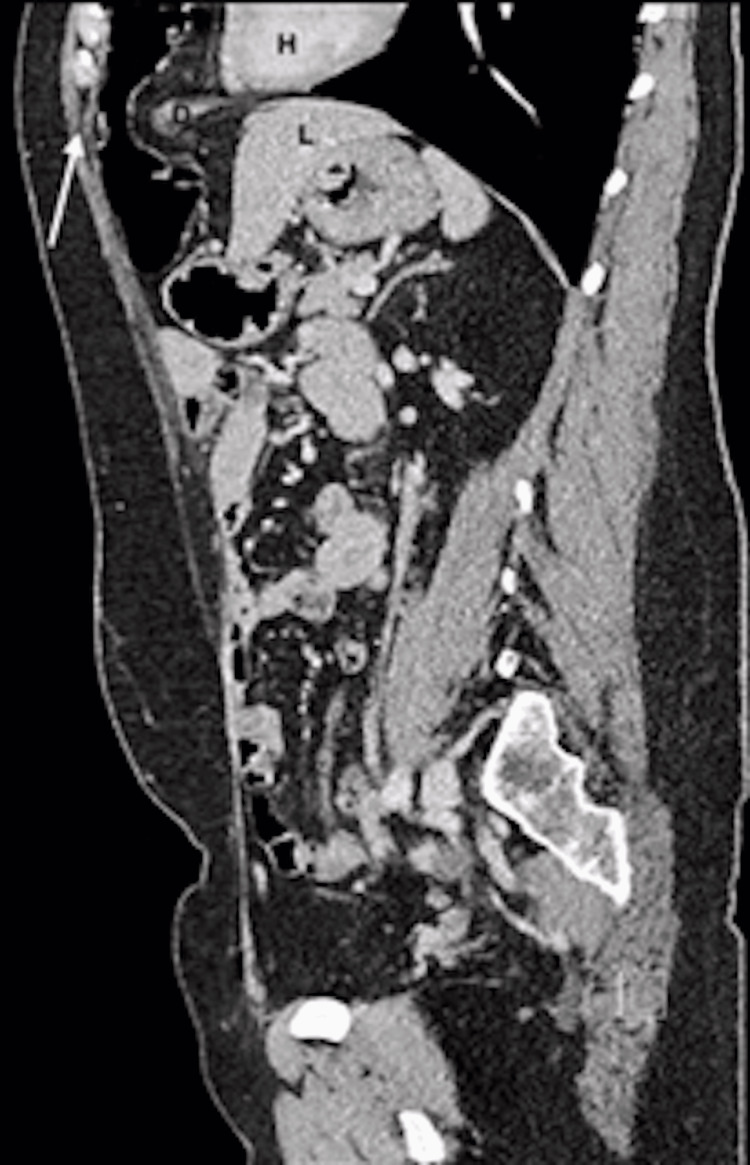
Sagittal view of abdominal and thoracic CT scan The white arrow points to the large left anterior diaphragmatic hernia with the passage of the transverse colon. H: Heart, D: Diaphragm, L: Liver

In 2021, a 42-year-old woman was referred to our digestive surgery department due to complaints of postprandial pain, nausea, vomiting, and dyspnea. The pain was described as a stabbing sensation radiating to the left breast and flank, with a feeling of imminent death. The pain was relieved by assuming the fetal position and pushing on the left flank. Upon abdominal examination, we observed a non-distended abdomen with tenderness in the left flank and normal bowel sounds. To further evaluate the patient's condition, we performed an abdominal and thoracic CT scan, which revealed a large left anterior diaphragmatic hernia containing a transverse colon and greater omentum, suggesting a Larrey’s hernia (Figures 1-3).

**Figure 4:**
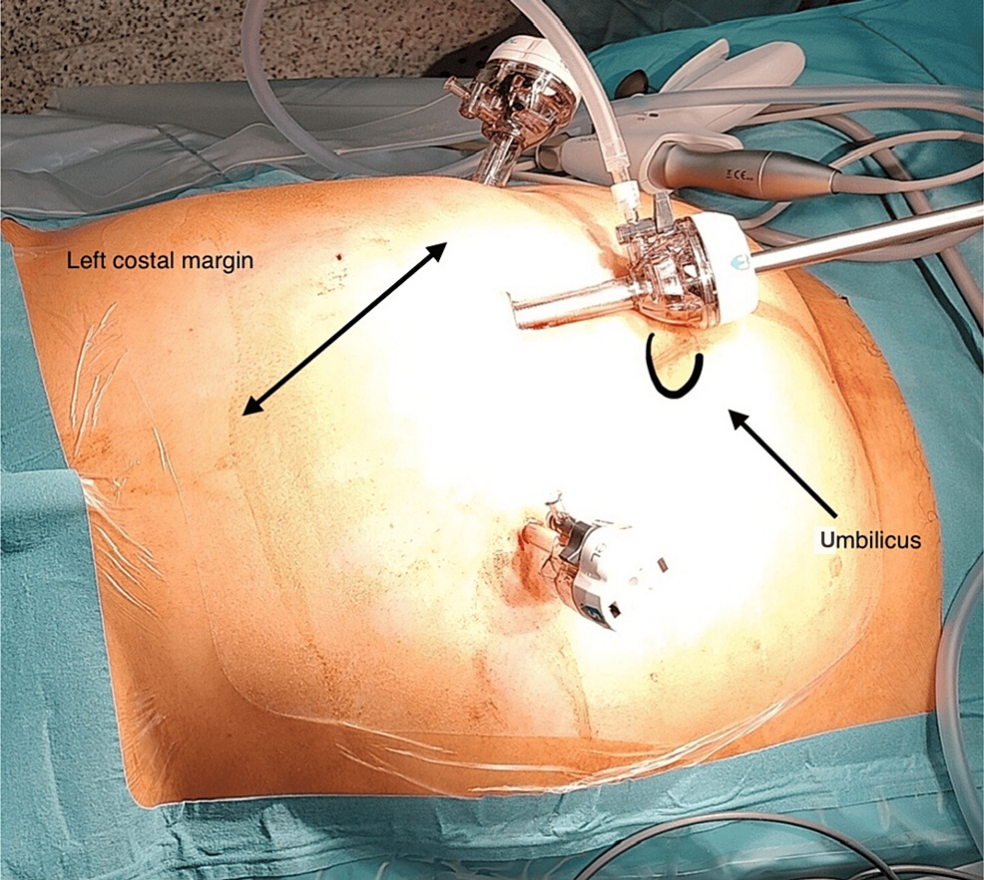
Three trocars were used as shown First, the 11-mm trocar was placed above the umbilicus for the scope, followed by the 5-mm trocar in the right flank and the 12-mm trocar in the left flank as working ports.

**Figure 5:**
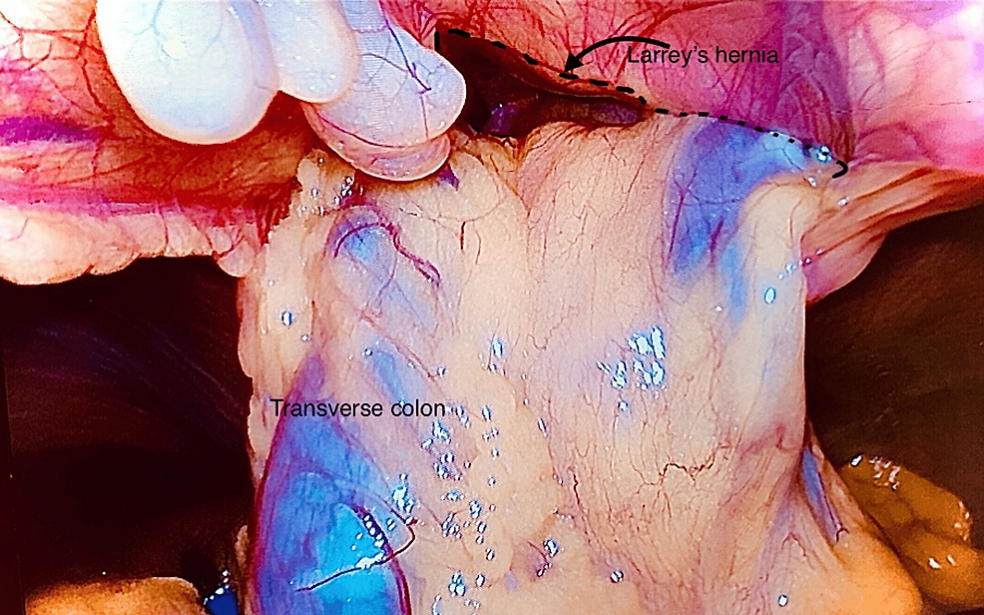
The progressive reduction of the transverse colon from the Larrey’s hernia

**Figure 6:**
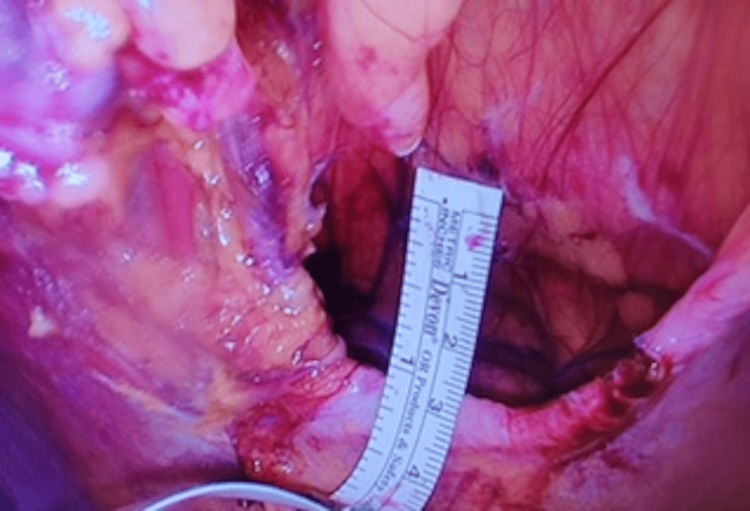
The defect after the reduction measuring 6 cm in length, 3 cm in width, and 10 cm in height

**Figure 7:**
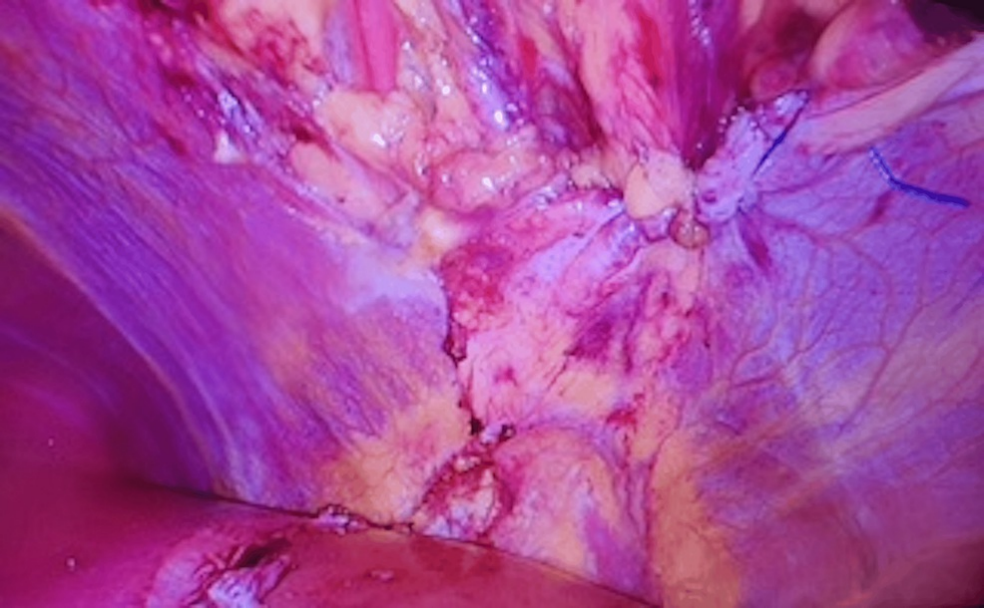
Intraoperative picture of the defect being closed by the V-lock

**Figure 8:**
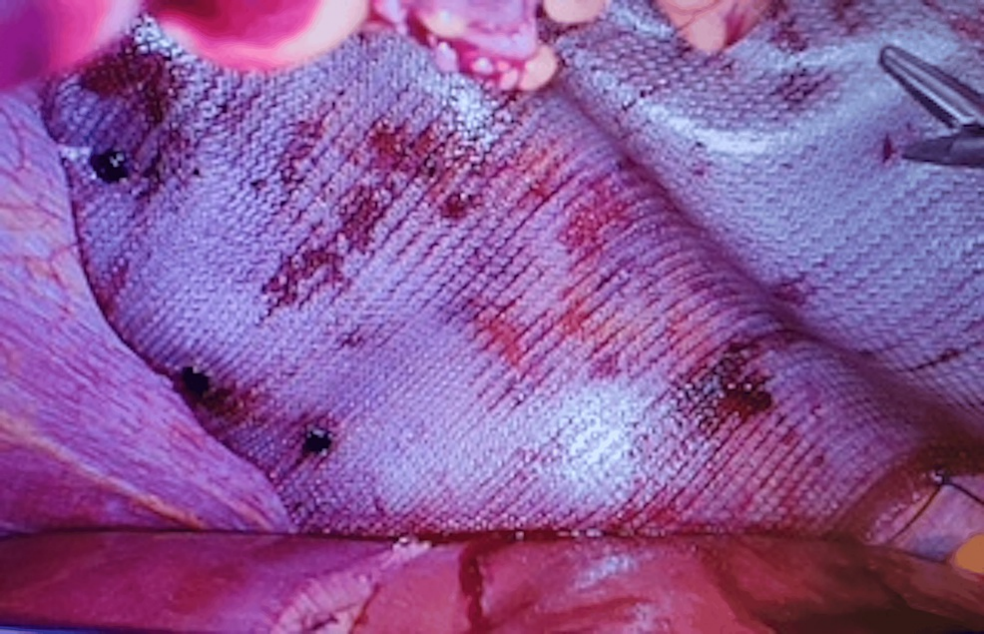
The Ventralight ST mesh in place

As the patient was symptomatic, our team decided to treat the hernia through a laparoscopic approach. The patient was placed in a supine position with legs apart and a bolster elevating the thorax. The pneumoperitoneum was created with a Veress needle in the left hypochondrium. Three trocars were inserted: one 11-mm trocar was placed above the umbilicus for the 10/30° scope, one 5-mm trocar in the right flank, and one 12-mm trocar in the left flank, both used as working trocars (Figure 4). The diaphragmatic hernia containing the transverse colon and greater omentum was identified and reduced (Figures 5-6). The falciform ligament was dissected. Adhesions at the entry orifice were released, and the sac was scarified. The defect was closed with a non-absorbable V-Loc® 2/0 suture (Medtronic, Minneapolis, MN, USA) (Figure 7), and a Ventralight ST Mesh Echo PS® (Becton Dickinson, Franklin Lakes, NJ, USA) with a diameter of 15 cm was applied, partially fixed with Sorbafix® (Becton Dickinson) and a Vicryl® 0 (Ethicon Inc., Raritan, NJ, USA) suture (Figure 8).

**Figure 9:**
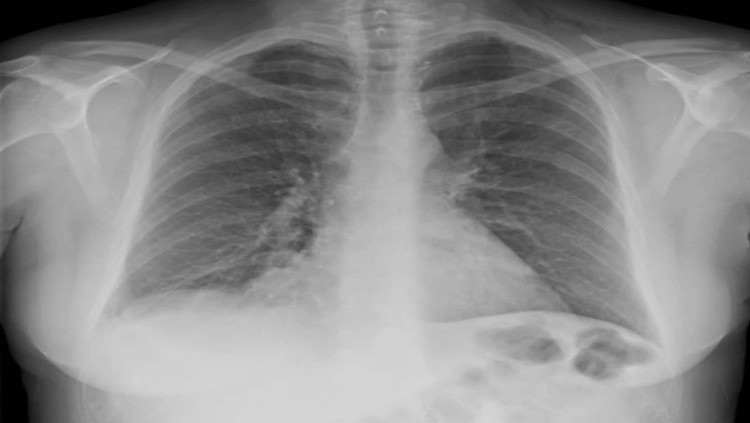
Immediate postoperative chest X-ray was normal

**Figure 10:**
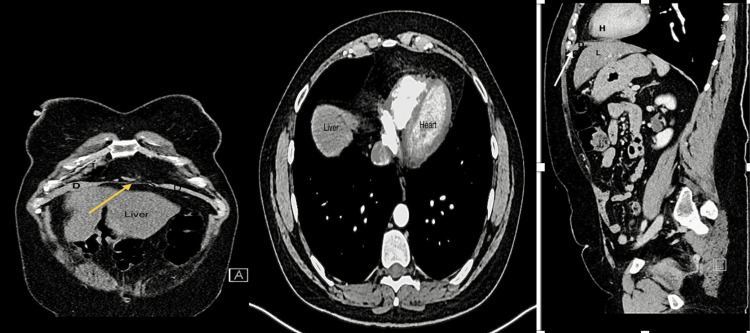
Abdominal and thoracic CT scan at six months shows no sign of diaphragmatic hernia recurrence (yellow and white arrow) D: Diaphragm, H: Heart, L: Liver

In the postoperative period, the patient did well, and a control chest X-ray was normal (Figure 9). She was discharged home 24 hours after the surgery. Ten days later, the patient underwent a follow-up assessment, which revealed a satisfactory postoperative recovery. Six months after the surgery, an abdominal and thoracic CT scan confirmed the absence of a recurrence of diaphragmatic hernia (Figure 10).

## Discussion

Morgagni-Larrey hernias are caused by a failure of the pars tendinalis part of the costochondral arches to fuse with the pars sternalis [3]. A systematic review showed that the average age of diagnosis for these hernias is 53 years [4]. In this study, the women had a rather normal distribution, with 15% presenting between 56 and 60 years of age. Our patient was 42 years old, a little young compared to the average. The diagnosis of Morgagni-Larrey hernias is often made in adulthood due to the lack of symptoms of this type of hernia.

A recent retrospective study showed that 19% of patients were asymptomatic, 36% had respiratory symptoms such as dyspnea and chest pain, 29% had gastrointestinal symptoms, and 17% had both [5]. Our patient belonged to the last category, mainly complaining of postprandial pain, nausea, vomiting, and dyspnea. These symptoms can be well explained by the contents of the hernia sac, which is mostly composed of the colon and the greater omentum [5]. The pain was described as a stabbing sensation radiating to the left breast and flank, with a feeling of imminent death. This could be due to postprandial increased intracolic pressure compressing the heart and lungs. And, by adopting the fetal position and pushing on the left flank, the patient tried to reduce the sac content and decrease the intraluminal and intrathoracic pressure.

Morgagni-Larrey has been associated in infancy or early childhood with other congenital anomalies, with the incidence ranging from 34% to 50% [3]. These anomalies include cardiac defects, trisomy 21, malrotation, anorectal malformations, omphalocele, skeletal anomalies, and the pentalogy of Cantrell [3,4]. Our adult patient did not have any of these conditions. Due to the symptoms of our patient, we directly performed an abdominal and thoracic CT scan, which yielded the diagnosis of Larrey’s hernia. In the literature, the defect is best identified by a CT scan, which has a 100% rate of correct diagnosis [3]. Even if the standard treatment has not been established for Morgagni-Larrey hernias, it is recommended that all Morgagni hernias be surgically repaired due to the risk of incarceration [3].

Because of its rarity, there are no well-established surgical techniques for the treatment of Morgagni-Larrey hernia [4]. It can be treated either through a transthoracic approach or a transabdominal approach. In the initial cases of Morgagni-Larrey hernias, surgery was performed using an open approach [4]. Nowadays, the use of minimally invasive techniques is increasing. For the abdominal approach, this technique facilitates an easier reduction of the hernia. It allows for the assessment of contralateral defects and the identification of any other abdominal pathologies [6]. In fact, there is a case reported in the literature where the patient was initially treated with thoracotomy for the hernia and presented two years later with intestinal obstruction due to a contralateral diaphragmatic hernia [7]. The main advantage of the thoracic approach is that it allows for easier dissection of the hernia sac from the mediastinal and pleural structures [8]. However, it requires single-lung ventilation, does not permit inspection of both sides of the diaphragm, and limits the ability to evaluate the contents of the hernia [9].

There is currently no consensus regarding Morgagni-Larrey hernias in terms of outcomes. However, the abdominal approach offers a distinct advantage in cases where strangulation or perforation is suspected [6]. The growing popularity of laparoscopic and thoracoscopic approaches as minimally invasive procedures has been documented to result in reduced surgical trauma, decreased postoperative pain, shorter hospital stays, and earlier resumption of activities compared to traditional open approaches in Morgagni-Larrey hernia cases [10]. Additionally, a case series has shown that minimally invasive surgery is associated with a lower incidence of chest tube usage [11].

The role of mesh reinforcement following the primary suture of hernia defects is still a matter of debate. There is currently no consensus on this matter. The three possible surgical treatment options for extra-hiatal diaphragmatic hernia are primary suture alone, primary suture with mesh reinforcement, or mesh interposition to reconstruct the diaphragmatic integrity without primary closure of the hernia by sutures [12]. In our case, we did not resect the hernia sac, and we opted for the closure of the defect (length 6 cm x width 3 cm x height 10 cm) with a non-absorbable V-Loc 2/0 suture and the placement of a Ventralight ST mesh with a diameter of 15 cm. The V-Loc 2/0 was very easy to use by laparoscopy. In the review by Horton, the average hospital stay after laparoscopy repair was three days. Our patient was very comfortable and left the hospital after 24 hours.

## Conclusions

Morgagni-Larrey hernia is a rare condition requiring surgical intervention. Diagnosis often occurs in adulthood, and laparoscopic repair offers favorable outcomes. Our case contributes to the understanding of this condition and supports the efficacy of laparoscopic management. Further research is needed to establish optimal treatment approaches for this rare pathology.
